# Exploring the Multiple Roles of Notch1 in Biological Development: An Analysis and Study Based on Phylogenetics and Transcriptomics

**DOI:** 10.3390/ijms25010611

**Published:** 2024-01-03

**Authors:** Yuesi Zhou, Zihao Yan, Ya Pang, Yao Jiang, Ruyu Zhuang, Shuyuan Zhang, Ayqeqan Nurmamat, Min Xiu, Ding Li, Liang Zhao, Xin Liu, Qingwei Li, Yinglun Han

**Affiliations:** 1 Key Research Base of Humanities and Social Sciences of Ministry of Education, Institute of Marine Sustainable Development, Liaoning Normal University, Dalian 116029, China; 15724341619@163.com; 2 Lamprey Research Center, College of Life Sciences, Liaoning Normal University, Dalian 116081, China; 15382241237@163.com (Z.Y.);; 3 Collaborative Innovation Center of Seafood Deep Processing, Dalian Polytechnic University, Dalian 116034, China

**Keywords:** Notch family, evolution, phylogeny

## Abstract

At present, there is a research gap concerning the specific functions and mechanisms of the *Notch* gene family and its signaling pathway in jawless vertebrates. In this study, we identified a Notch1 homologue (*Lr.* Notch1) in the *Lethenteron reissneri* database. Through bioinformatics analysis, we identified *Lr.* Notch1 as the likely common ancestor gene of the *Notch* gene family in higher vertebrates, indicating a high degree of conservation in the *Notch* gene family and its signaling pathways. To validate the biological function of *Lr.* Notch1, we conducted targeted silencing of *Lr*. Notch1 in *L. reissneri* and analyzed the resultant gene expression profile before and after silencing using transcriptome analysis. Our findings revealed that the silencing of *Lr.* Notch1 resulted in differential expression of pathways and genes associated with signal transduction, immune regulation, and metabolic regulation, mirroring the biological function of the Notch signaling pathway in higher vertebrates. This article systematically elucidated the origin and evolution of the *Notch* gene family while also validating the biological function of *Lr.* Notch1. These insights offer valuable clues for understanding the evolution of the Notch signaling pathway and establish a foundation for future research on the origin of the Notch signaling pathway, as well as its implications in human diseases and immunomodulation.

## 1. Introduction

The discovery of the *Notch* gene can be traced back to 1917, when it was initially identified in Drosophila melanogaster [1]. The Notch signaling pathway is unique, as it transmits signals through receptor–ligand interactions between neighboring cells. It exerts its biological functions through interaction with other pathways and by regulating the transcription of downstream target genes [2]. The Notch signaling pathway primarily comprises four components: receptors, ligands, the DNA-binding proteins CSL (CBF-1, suppressor of hairless, Lag), and downstream target genes. In mammals, there are four Notch receptors (Notch1–4) and five typical ligands (JAG1, JAG2, DLL1, DLL3, DLL4). Upon activation, the Notch protein undergoes three proteolytic cleavages, releasing the intracellular domain (NICD) into the cytoplasm. The NICD then translocates to the nucleus, where it binds to the transcription factor CSL, forming the NICD–CSL transcription activation complex. This complex activates downstream target genes such as *HES*, *HEY*, *ERBB2*, and *VEGFR*, enabling the Notch signaling pathway to perform its biological functions [3,4,5]. In higher vertebrates, the Notch signaling pathway plays a crucial role in regulating cell fate during both embryonic development and adulthood [6,7]. Disruption of Notch signaling often leads to embryonic mortality [8]. In progenitor and stem cells, the Notch signaling pathway promotes differentiation and self-renewal, preserves the stemness of progenitor cells as well as the stem cell bank, and directs lineage commitment in progenitor cells [9].

In addition, aberrant Notch signaling pathways have been implicated in various cancers and non-cancerous diseases. It is now known that dysregulation of the Notch signaling pathway occurs in various tumors as well as several non-cancerous conditions, such as leukemia, kidney cancer, bladder cancer, breast cancer, etc. [10]. Studies have shown that the Notch1 mutation suppresses the metastasis of esophageal cancer cells, thereby potentially impeding the malignant transformation of esophageal cancer. Conversely, activating Notch1 increases the susceptibility to esophageal cancer occurrence [11,12,13,14]. Through its interaction with molecules like *LEF1* and *Cox-2*, Notch2 enhances resistance to chemotherapeutic agents, which in turn influences the prognostic response to chemotherapy [15]. Furthermore, recent studies have shed light on the impact of Notch2 on the development and metastasis of lung cancer [16,17]. Notch3 has been found to play a protumorigenic role in numerous cancers, including vascular tumors, T-cell lymphoma, nasopharyngeal carcinoma, breast carcinoma, and ovarian carcinoma, among others [18]. Unlike other Notch receptors, Notch4 exhibits distinct functions in different types of cancer, and blocking *Notch4* activation could potentially affect the functioning of Notch1–3 receptors. Therefore, comprehending the interplay between Notch4 and other Notch receptors holds significant importance for research pertaining to the *Notch* gene family and the evolutionary aspects of the Notch signaling pathway [19].

As the direct ancestors of vertebrates, agnathans played a crucial role in the origin and evolution of vertebrates. Among them, the lamprey, one of the oldest existing agnathans, is considered an ideal model organism for research in genetic, physiological, and immunological domains due to its simple structure and evolutionary conservatism [20]. Recent discoveries regarding variable lymphocyte receptors (VLRs) in lampreys have opened up avenues for studying the evolution of the adaptive immune system in vertebrates [21,22]. Unlike higher vertebrates, agnathans lack the same adaptive immune system but possess an alternative adaptive immune system that utilizes variable lymphocyte receptors (VLRs) to bind with antigens [23,24]. In lampreys and hagfish, three typical VLR genes, namely *VLRA*, *VLRB*, and *VLRC*, have been identified [25,26,27]. VLRB-expressing cells in these species resemble mammalian B cells, while VLRA- and VLRC-expressing cells resemble mammalian T cells [28]. Furthermore, a recent study has discovered two new *VLR* genes, *VLRD* and *VLRE*, in the lamprey [10], and further investigations are currently underway. From an evolutionary perspective, this study sheds light on the intricate immune processes in humans by conducting comparative studies on the adaptive immune systems of jawless and jawed vertebrates, providing a deeper understanding of these processes [29].

Although there is extensive research on the Notch signaling pathway in higher vertebrates, little has been conducted on jawless vertebrates. This study aimed to address this gap by identifying and cloning the *Notch1* gene from the genome database of *L. reissneri* [30]. Subsequently, we employed bioinformatics analysis to investigate the evolutionary origin of the *Notch* gene family and confirmed *Lr.* Notch1 as the ancestral gene of the entire *Notch* gene family. Moreover, our research revealed the involvement of the Notch signaling pathway in various biological functions, including signal transduction, oxygen regulation, and immune regulation in the lamprey. We discovered that lampreys possess the capability to modulate the Notch signaling pathway in conjunction with other signaling pathways, including Wnt, HIF-1, and mTOR. By utilizing the lamprey as a model organism, we aimed to unravel the intricate pathogenic mechanisms of the Notch signaling pathway, starting from its simple structure. This study lays a solid foundation for investigating the implications of the Notch signaling pathway in human diseases.

## 2. Results

### 2.1. Phylogenetic Analysis of Notch1

#### 2.1.1. Identification and Evolutionary Analysis of the Notch Members in the Genome Database of the *Notch* Gene Family

**Figure 1:**
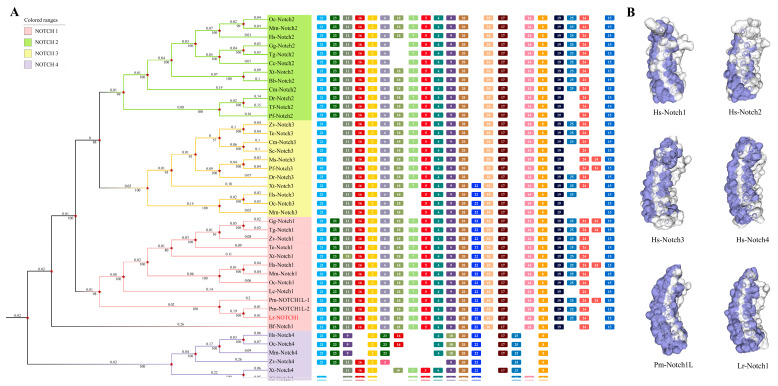
Bioinformatics Analysis. The identification and sequence analysis of Notch family genes. (**A**) The phylogenetic tree for the Notch family based on the NJ method. An NJ tree was constructed using the amino acid sequences of the Notch proteins. The four branches of Notch1-4 are highlighted in pink, green, yellow and purple, respectively. The red font in the figure represents Notch1 in lampreys. “★” represents a branch node. The numbers at the nodes indicate the bootstrap confidence values derived from 1000 replications. A motif composition of the Notch family proteins. The motifs, which are numbered 1–25, are shown in different colored boxes. The full names of species and the corresponding accession numbers of Notch proteins are listed in Supplementary Table S1. The matching amino acid sequences in motifs are listed in Supplementary Table S2. (**B**) Tertiary structure prediction of Notch1, Notch2, Notch3, and Notch4 from humans and lampreys. The white represents a random coil; the green represents a β-fold; and the purple represents an α-helix.

By analyzing the amino acid sequences of Notch1–4 genes of higher vertebrates from the lamprey genome database and the sea lamprey genome database (Table S1), we performed a homology analysis to elucidate the origin and evolution of the entire *Notch* gene family. Our analysis revealed a *Notch* gene family member in *L. reissneri* that exhibited similarity to Notch1 in other, higher vertebrates. Through multiple sequence comparisons, a high degree of similarity was found between these genes, leading us to designate this gene *Lr.* Notch1. To further investigate the evolutionary relationships within the *Notch* gene family, we constructed a phylogenetic tree using the NJ method, (Figure 1A) The phylogenetic analysis demonstrated that *Lr*. Notch1 clustered with Notch1 from other species and served as an outgroup for Notch1. Notch1 appeared on the periphery and grouped with Notch2/3. Conserved motifs, including motifs 3, 8, and 20, were present in all *Notch* genes, including in the lamprey and hagfish Notch1. Additionally, motifs 4 and 21 were relatively conserved and found in almost all *Notch* genes. We further analyzed and compared the motifs of all Notch homologs included in the phylogenetic tree (Figure 1A; Table S2). Motifs 22, 23, and 24 were simultaneously present in Notch1 and lamprey Notch1, while Notch2 lacked motif 22, and Notch3 lacked motif 23. Notch4 exhibited notable differences, lacking motifs 6, 19, 15, and 10 and showing a significant alteration in the position of motif 25. These findings suggest that Notch4 has undergone considerable evolutionary changes, while motifs in Notch1/2/3 have been relatively preserved.

**Figure 2:**
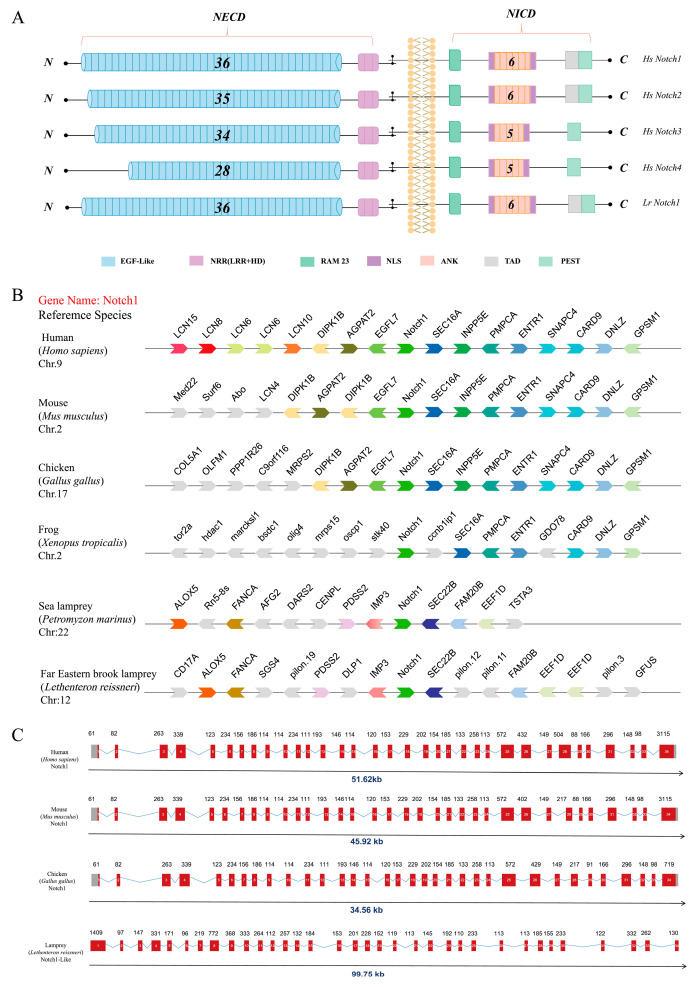
Domains, syntenic relationships, and gene structure in the Notch family. (**A**) A comparison of the domains between Notch1, Notch2, Notch3, Notch4 from humans and lampreys. (**B**) The conservation of genes neighboring Notch family genes. The genes are indicated by arrowheads. The arrows and arrowheads pointing in opposite directions indicate genes located on opposite strands. Chr denotes the chromosome. Different colors represent different genes. (**C**) A representation of the available sequence data for the *Notch* family genes. Noncoding exons are designated with open boxes, and coding exons are indicated by colored boxes. The transcription start site is indicated by an arrow. Notch1 in different species consists of 34 exons, which are represented by numbers 1–34 in the diagram.

Based on the aforementioned data, the evolutionary status of lampreys suggests that *Lr*. Notch1 may be more primitive and potentially serve as the ancestral gene for vertebrate Notch1. Comparative analysis of the domain and three-dimensional (3D) structure of Notch1 protein in lampreys with the Notch protein family in jawed vertebrates supports the notion of relative conservation (Figure 1B). The functional structural domains of *Lr*. Notch1 are also preserved (Figure 2A), similarly to mammalian Notch1, encompassing 36 EGF-like domains, three NRR domains, six classical ANK domains, an RBP-Jκ-Associated Molecule (RAM) domain, and a proline-glutamic acid-serine-threonine (PEST) domain. However, it is worth noting that while lampreys possess the trans-activating structural domain (TAD) in Notch1, higher vertebrates only exhibit this domain in Notch1/2 [31]. Furthermore, there are some differences between Notch1 and Notch3/4 in terms of the quantity of ANK and EGF-like structural domains in lampreys, highlighting further evidence of Notch1’s evolutionary conservation.

#### 2.1.2. Analysis of Collinearity and Gene Structure of the *Notch1* Gene in the Lamprey

The establishment of gene homology is facilitated by the study of genomic collinearity, which contributes to a better understanding of the evolutionary relationships among members of gene families [32]. In this study, we conducted collinearity analyses on the *Notch1* gene in *L. reissneri* and Petromyzon marinus, as well as in humans, mice, and chickens, with the aim of enhancing our comprehension of vertebrate evolutionary history (Figure 2B). Our analysis results reveal that downstream genes, particularly those associated with the Notch1 gene and its adjacent genes, are relatively conserved in other jawed vertebrates. The *Notch1* gene forms a distinct chain cluster with its adjacent genes. Conversely, the two species of lampreys exhibit a similar linear relationship between the upstream and downstream adjacent genes of the *Notch1* gene, but their associated neighboring genes differ significantly from those of jawed vertebrates. Consequently, we postulate that during the lengthy evolutionary process from lampreys to humans, events such as homologous recombination or chromosomal translocation may have led to alterations in the arrangement pattern of genes. Gene structure analysis results (Figure 2C) demonstrate that the *Notch1* gene in lampreys shares a comparable exon and intron structure with other representative species, consisting of a total of 34 exons. This finding supports our hypothesis and further underscores the conserved evolutionary status of Notch1.

Taken as a whole, our findings suggest that *Lr.* Notch1 might serve as the ancestral gene for the *Notch* gene family in higher vertebrates. Throughout the course of development, this ancestral gene underwent intricate modifications through adaptive evolution, giving rise to distinct members such as Notch1, Notch2, Notch3, and Notch4.

### 2.2. Tissue Distribution, Immune Response, and siRNA-Mediated Specific Gene Silencing

#### 2.2.1. Distribution of *Lr.* Notch1 in Various Tissues of *L. reissneri* and siRNA-Mediated Specific Gene Silencing

**Figure 3:**
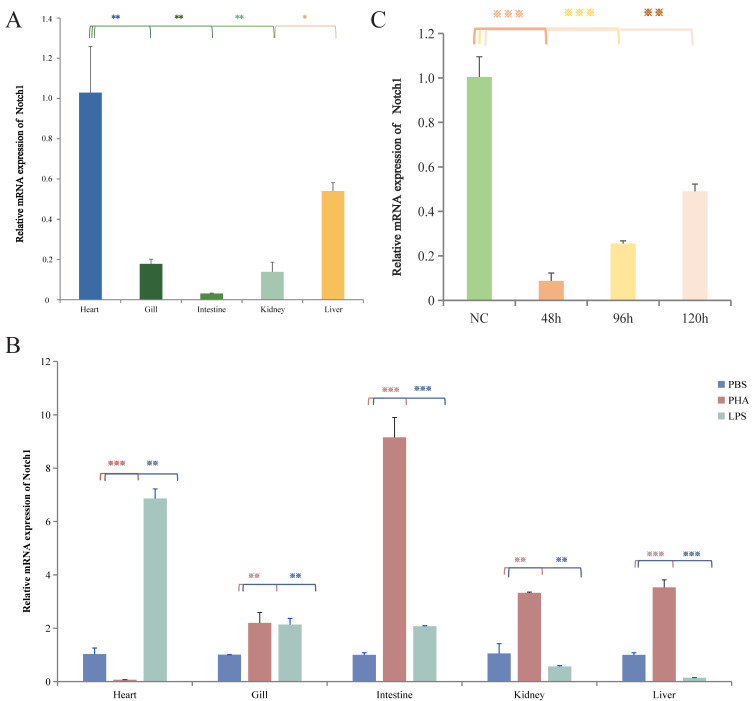
Distribution, immune response, and siRNA-mediated specific gene silencing of *Lr.* Notch1. (**A**) Relative *Lr.* Notch1 mRNA expression levels in various lamprey tissues. Different colors represent different tissues. (**B**) Relative mRNA expression levels of *Lr.* Notch1 in lamprey responses to PHA and LPS. PBS was used as a blank control. PBS, PHA, and LPS are respectively represented by the colors blue, red, and green. (**C**) The expression of the *Lr.* Notch1 gene after *Lr.* Notch1 gene silencing was assessed by qPCR, and the abundance map was drawn. Different colors represent different time periods. L-GAPDH was used as the reference gene, and the experimental results were expressed as means ± SD. * Indicates a significant difference compared with the control group (*n* = 3, Ns: not significant, * *p* < 0.05, ** *p* < 0.01, *** *p* < 0.005).

Using the qPCR technique, we successfully detected the expression of *Lr.* Notch1 in various tissues, including the heart, gills, intestine, kidney, and liver tissues of *L. reissneri* (Figure 3A; Table S3). Notably, amplification of the *GAPDH* gene in all tissues confirmed the validity of the individual templates. Among these tissues, the highest expression of *Lr.* Notch1 was observed in the cardiac tissue of healthy larval Anguilla rostrata, followed by the liver tissue, while the intestinal tissue exhibited the lowest expression. This expression pattern aligns with the known expression profile of Notch1 in higher vertebrates [33]. The coordinated development of the heart, an organ in which the Notch signaling pathway plays a crucial role, necessitates interactions at multiple sites. Numerous functional studies in vertebrates have demonstrated the indispensable role of Notch as a key molecule in cardiovascular development, and mutations in certain components of the Notch signaling pathway have been implicated in congenital cardiovascular diseases in humans [33,34].

In order to further investigate the biological function of the *Lr.* Notch1 gene in the mudskipper, based on the expression results for *Lr.* Notch1 in various tissues of *L. reissneri*, we conducted targeted silencing of *Lr.* Notch1 (Table S4) to gain insights into the biological function of this gene in the seven-gilled shark. Subsequently, we employed qPCR technology to assess the silencing effect at different time intervals (Figure 3C). The impact of silencing became more pronounced over time, with a significant decrease in the expression of *Lr.* Notch1 observed starting at 48 h. However, a gradual recovery was observed at 96 h. Consequently, the heart tissue was selected for transcriptome sequencing 48 h after silencing to further investigate the effects of *Lr.* Notch1 silencing.

#### 2.2.2. The Immune Response to Various Stimuli Mediated by *Lr.* Notch1

Previous investigations have elucidated the involvement of Notch in cell fate determination and complex immunological responses in higher organisms. In higher vertebrates, the Notch signaling pathway intersects with NF-κB, PI3K-AKT, and other signaling pathways, as well as regulating molecules such as HES, HEY, CXCR4, IL-4, and IL-7 to orchestrate diverse immunological functions [35,36,37]. For example, the Notch signaling pathway promotes invasion, self-renewal, and proliferation of glioma-starting cells via controlling the chemokine system CXCL12/CXCR4 [35]. Additionally, studies have indicated that the interaction between Notch and TLR signals can induce the expression of Notch target genes *Hes* and *Hey*, resulting in augmented production of TLR signaling activation products, including tumor necrosis factor alpha (TNF-α), interleukin 6 (IL-6), and IL-12 [38,39]. In addition, it was discovered that LPS stimulated TLR4 and Jag1/Notch1, which in turn triggered the NF-κB signaling pathway, resulting in an inflammatory cascade. Furthermore, the two signaling pathways, TLR4-NF-κB and Notch1-NF-κB, encourage each other’s inflammatory responses through LPS. Notably, blocking Notch signaling significantly attenuates the inflammatory signaling responses induced by LPS [40]. In the Leptobotia elongata genome database, we have also identified the aforementioned immune molecules; however, further research is required to elucidate the interactions between these molecules and *Lr.* Notch1.

Lipopolysaccharide (LPS) and phytohemagglutinin (PHA) are mitogens for B cells and T cells in higher vertebrates, respectively. Previous studies have shown that *Lethenteron camtschaticum* exhibits upregulated expression of VLRA following stimulation with PHA. After LPS stimulation, the expression level of VLRB is upregulated, indicating that both PHA and LPS can induce immune response in the lamprey [41]. To investigate the role of *Lr.* Notch1 in the immune response of lamprey, we stimulated healthy individuals with two groups of stimuli, PHA and LPS. The results showed that *Lr.* Notch1 responded to different mitogens in all tissues (Figure 3B). In the intestine, gills, kidneys, and liver tissues, the response to PHA stimulation was observed, while in the heart, gills, and intestine, the response to LPS stimulation was observed. These findings suggest a potential correlation between *Lr.* Notch1 and the immune response in *L. reissneri*.

### 2.3. Analysis of Transcriptional Data from Lr. *Notch1* Gene Silencing

The experimental group (siNotch) and the control group (NC) were subjected to transcriptomics analysis, with three biological replicates performed for each group. The obtained results included mRNA differential expression profiles and enrichment analysis. Following screening for differentially expressed genes (DEGs) with a *p* value < 0.05 and a fold change ≥ 2, a total of 431 DEGs were identified. Among these, 169 genes were found to be up-regulated, representing 39.2% of the total number of DEGs, while 262 genes were down-regulated, accounting for 60.8% of the DEGs (Figure S1).

#### 2.3.1. Enrichment Analysis of Differentially Expressed Genes

**Figure 4:**
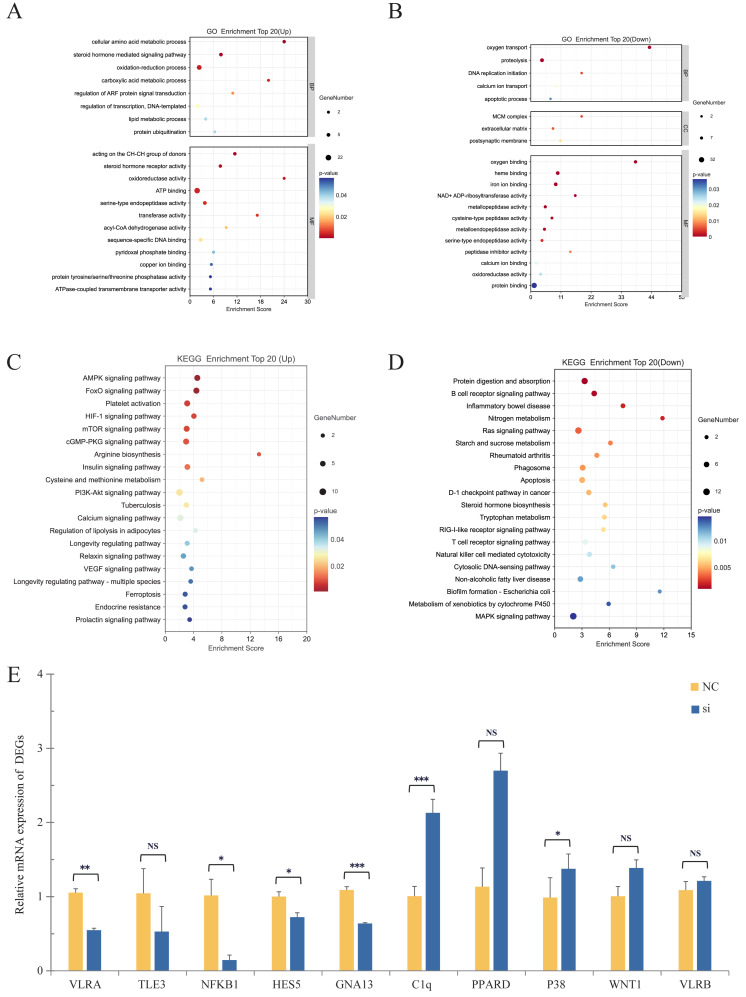
*Analysis of Lr. Notch1 Gene Silencing Transcriptome Data***.** (**A**) Go enrichment analysis results for *Lr.* Notch1 gene silencing transcriptome up-regulated expression genes. (**B**) Go enrichment analysis results for *Lr.* Notch1 gene silencing transcriptome down-regulated expression genes. (**C**) KEGG enrichment analysis results for *Lr.* Notch1 gene silencing transcriptome up-regulated expression genes. (**D**) KEGG enrichment analysis results for *Lr.* Notch1 gene silencing transcriptome down-regulated expression genes. (**E**) Bar chart for detection of relative expression of differential genes by Q-PCR in the *Lr.* Notch1 transcriptome. Ns: not significant, * *p* < 0.05, ** *p* < 0.01, *** *p* < 0.005 (paired *t*-test).

We conducted GO and KEGG enrichment analyses on the differentially expressed genes (DEGs) obtained from the silenced group and the NC group. Results with a minimum number of enriched differential genes (listhits) ≥ 2 were selected using a screening criterion of *p* < 0.05, and the top 20 bubble plots enriched with up-regulated and down-regulated DEGs were visualized (Figure 4A,B). These analyses revealed that the DEGs mainly encompass two major categories in the GO analysis: biological processes and molecular functions, with less involvement in cellular components. The biological process module demonstrated that the up-regulated DEGs were primarily enriched in cellular amino acid metabolism, oxygen transport, and oxidation–reduction processes, among others, while the down-regulated DEGs were primarily enriched in oxygen transport and signal transduction, among others. In contrast, in the molecular function module, the up-regulated DEGs were primarily enriched in steroid hormone receptor activity and serine-type endopeptidase activity, among others, while the down-regulated DEGs were mainly enriched in oxygen binding, heme binding, and iron ion binding, among others. The KEGG enrichment results indicated that the up-regulated DEGs were primarily concentrated in pathways related to amino acid metabolism, carbohydrate metabolism such as tryptophan metabolism, arginine biosynthesis, glycolysis/gluconeogenesis, etc. (Figure 4C), whereas the down-regulated DEGs were mainly abundant in pathways related to signal molecule interactions, immune diseases, signal transduction, such as significantly associated rheumatoid arthritis, the IL-17 signaling pathway, and the PI3K-Akt signaling pathway, among others. (Figure 4D).

Integration of the findings from the GO and KEGG analyses revealed that silencing Notch1 in lamprey had significant effects on organismal metabolism, signal transduction, enzyme activity regulation, and self-oxygen regulation. Notably, silencing *Lr*. Notch1 resulted in the up-regulation of several genes encoding metabolic-related enzymes, including glutamate dehydrogenase, glycine dehydrogenase, succinyl-CoA dehydrogenase, and acetyl-CoA carboxylase. Consequently, the activities of these enzymes were enhanced, leading to increased ATP-binding capacity and promoting the activity of metabolic and oxygen transport pathways. This suggests that *Lr*. Notch1 may play a role in organismal metabolic pathways. Silencing *Lr*. Notch1 induced a stress state in the organism, activating compensatory mechanisms and accelerating redox reactions, ultimately increasing the metabolic rate. Conversely, the down-regulation of molecules involved in signal transduction, such as Guanine nucleotide-binding protein subunit alpha-13 (Gα13), Rho family GTPase 1 (RhoA), Signal Transducer and Activator of Transcription (STAT), and RasGTPase, hindered the corresponding signaling pathways, resulting in a deceleration of signal transduction. Furthermore, besides its role in hematopoiesis and blood oxygen regulation, hemoglobin (HB) expression was affected by Notch1 silencing, indicating that Notch1 is not only crucial in signal transduction but also participates in the regulation of multiple signaling pathways and organismal growth and development in the mudskipper.

Our transcriptome data revealed differential expression of molecules related to the aforementioned pathways, such as PI3K, Wnt, RhoA, ECM, TLR2, TNF, and FZD2, which further demonstrates the close relationship between the Notch signaling pathway and these pathways in *L. reissneri*. This finding underscores the conservation of the Notch pathway in regulating signaling pathways.

#### 2.3.2. Validation of RNA-Seq Results by qPCR

To validate the RNA-Seq data (Table S5) obtained in this study for both the NC control group and the Notch1-silenced group, we utilized GAPDH as an internal reference. Ten randomly selected genes enriched in the GO and KEGG pathways, as well as differentially expressed genes (DEGs) closely associated with the Notch signaling pathway, were validated using quantitative polymerase chain reaction (qPCR). The results demonstrated consistent expression patterns between the qPCR data and the transcriptome data (Figure 4E), confirming the reliability of the transcriptome data generated in this study.

**Figure 5:**
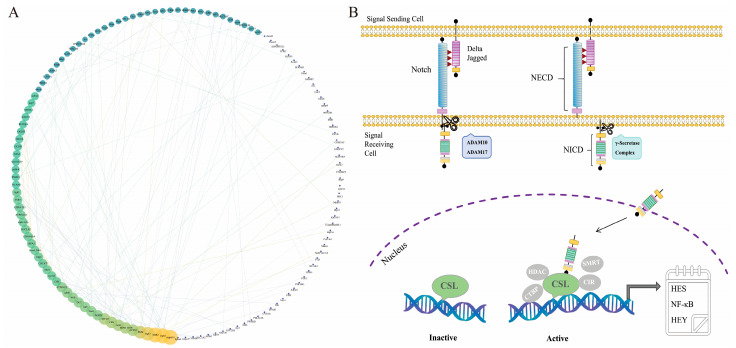
PPI network and Notch signaling pathway of Lamprey (**A**) Protein–protein interaction (PPI) network of *Lr.* Notch1 RNA-seq. The size of the two graphs indicates the number of interacting proteins and using a color gradient from yellow to blue. The width of the lines indicates the protein interaction evaluation score. (**B**) Notch signaling pathway of lamprey. The Notch depicted in the figure is Notch1, while the information regarding other molecules is listed in Table S6.

Furthermore, after silencing *Lr.* Notch1, a blast analysis was conducted using the protein sequences of lampreys and sea lampreys available in the String database. The protein interactions of sea lampreys were mapped onto Lamprey’s ID, and the top 160 protein interaction results with a combined score greater than 650 were selected to construct a protein–protein interaction (PPI) network map (Figure 5A). In the PPI results, we observed that molecules such as MAPK14, ATP6V1, Itgbs, MB2, STAT3, PKM, ACSS3, and MYL9, which are associated with signal transduction, inflammation, and tumor invasion, exhibited more active interactions with other molecules. Among them, MAPK14 showed the widest range of interactions. These findings further suggest the correlation between *Lr.* Notch1 and the aforementioned molecules, as well as the potential role of *Lr.* Notch1 in signal transduction, inflammation, and tumor invasion. However, the specific mechanisms underlying the interactions of these related molecules remain to be further elucidated.

## 3. Discussion

In recent years, the Notch signaling pathway has garnered significant attention in the field of human disease development and therapy due to its involvement in numerous disorders [41,42]. Research on human diseases has revealed a cross-talk between the Notch signaling pathway and other signaling pathways, including Hedgehog, RAS, PI3K-AKT, Wnt, HIF-1, and mTOR, in various disorders [43,44,45]. 

This study effectively addressed the research gap concerning the origin and evolution of the Notch signaling pathway by identifying a *Notch1* gene in *Lethenteron reissneri*. Additionally, the findings suggest that *Lr*. Notch1 may serve as the direct ancestor of the *Notch* gene found in higher vertebrates, a proposition supported by phylogenetic analysis. This analysis also delineated the unique evolutionary position of Notch in higher vertebrates, encompassing the functions of at least three members of the Notch family. We found all members of the Notch signaling pathway, including Notch1, ADAM10/17, HDAC, HES, and others, in the *L. reissneri* genome database (Table S6). Subsequently, we reconstructed a comprehensive Notch signaling pathway in *L. reissneri* (Figure 5B), indicating that *L. reissneri* possesses a Notch signaling pathway similar to that of higher vertebrates, further elucidating the evolutionary conservation of the Notch signaling pathway. In addition, in the enrichment results, we also identified the association of *Lr.* Notch1 with numerous pathways related to human diseases such as rheumatoid arthritis, atherosclerosis, HPV infection, vascular smooth muscle contraction, tuberculosis, malaria, type 2 diabetes, and others. We observed differential expression of immune-regulating molecules such as *CXCR4*, *IL-17*, *NFKB1*, *TNF10*, *C3*, and *C1Q.* It implies that the Notch signaling pathway in lampreys plays a role in immune regulation and disease development similar to that in humans. Refining the study of the Notch signaling pathway in lampreys can, to some extent, simplify the research path for studying diseases in the complex mechanism network of humans. 

Previous studies have identified cytidine deaminase (*Lr*.CDA) as a key molecule involved in mediating class switch recombination (CSR) of lamprey VLRs. Recent studies have shown that CDA*2* in lampreys is involved in the assembly of VLRB [46,47]. Notably, in this study, we observed significant alterations in the expression levels of variable lymphocyte receptors (VLRs) in lampreys among the numerous differentially expressed genes (DEG*s*). Specifically, the expression of VLRA and VLRC was down-regulated, while VLRB expression was up-regulated. This also implies that *Notch1* may have a certain relationship with the adaptive immune response of lampreys. Interestingly, in our study, silencing *Lr*-Notch1 resulted in a trend of up-regulated expression of *Lr*-CDA1. This suggests that *Lr*-Notch1, VLRs, and CDA may have a specific role interconnected with each other, potentially being involved in the CSR process of lampreys. Further research is currently underway.

This study still has certain limitations: for example, the research is limited to the transcription level and has not yet conducted studies at the protein level; at the same time, there is a lack of research on the functions of other members in the Notch signaling pathway in lampreys. In the future, we plan to study the protein levels of the *Notch* gene family and all members of the Notch signaling pathway in lampreys in order to further explore the functions of the Notch signaling pathway in lower vertebrates. We also intend to investigate the role of *Notch* and its related molecules in the adaptive immune network mediated by VLRs in lampreys, with the hope of unraveling the mechanisms underlying adaptive immunity in lampreys. In addition, we also intend to delve into the mechanisms by which the Notch signaling pathway in lampreys contributes to immune responses. By studying lampreys from an evolutionary perspective, we hope to provide a simpler and more intuitive reference for the study of human diseases.

## 4. Materials and Methods

### 4.1. Experimental Animals

Lamprey larvae (*L. reissneri*) were procured from the Shuifen reservoir of the Yalu River, located in Dandong City, Liaoning Province, China, in April. A specialized laboratory facility was employed to recreate a natural benthic habitat conducive to lamprey maintenance. The study was conducted following ethical guidelines and received approval from the Animal Welfare and Research Ethics Committee of the Institute of Dalian Medical University (Permit Number: SYXK2004-0029). All experiments were performed in accordance with the approved guidelines. Euthanasia of the animals was carried out using 0.02% MS222.

### 4.2. Multiple Sequence Alignment and Phylogenetic Analysis

The amino acid sequences of all four members (Notch1–4) of the *Notch* gene family were searched for in the NCBI database for representative species ranging from mammals to cartilaginous fish, such as mouse and zebrafish. Additionally, the amino acid sequences of Notch family members from the sea lamprey *Pm*.Notch1 were obtained. The amino acid sequences of Notch family members from the sea lamprey *Lr.* Notch1, which were constructed in our laboratory, were compared with the homologous sequences of Notch family members from the sea lamprey *Pm*. Notch1. Multiple sequence alignment was performed using BioEdit 7.0.9 software on the obtained amino acid sequences. A phylogenetic tree was constructed using MEGA software (Version 10.0.1) with 1000 bootstrap replicates, employing the neighbor-joining (NJ) method.

### 4.3. Conservation Domain Prediction, Tertiary Structure Prediction

The amino acid sequences of *Lr*. Notch1 and human Notch1–4 were sequenced and analyzed in the conserved structural domain using the SMART online software (http://smart.emblheidelberg.de) Additionally, the online software Swiss-Model, (http://swissmodel.expasy.org/interactive), was utilized to predict the three-dimensional model of Notch1 protein from lamprey *Lr*. and human Notch1–4 proteins.

### 4.4. Gene Structure, Collinearity, and Motif Analysis

The Ensembl online software (http://asia.ensembl.org/index.html) was utilized to analyze the gene structure of the *Notch* gene family. The exonic and intronic positions of representative species, including humans, mice, and chickens, were retrieved. The genomic sequence and CDS sequence of the *Lr. Notch1* gene in the *L. reissneri* database were also downloaded and analyzed for intron and exon positions using the Gene Structure Display Server website. The resulting gene structure diagram was drawn using Adobe Photoshop 2020.

Gene synteny analysis was performed using the Genomicus online tool (Genomicus https://www.genomicus.bio.ens.psl.eu/genomicus-104.01) and the Ensembl database (http://asia.ensembl.org/index.html). Different colors were assigned to represent different genes, while orthologous genes were labeled with the same color. Relevant data from the lamprey database were used to perform gene synteny analysis for the lamprey.

Conserved motif analysis was performed by MEME 5.3.1 online software (https://meme-suite.org/meme/tools/meme) with the following parameters: zero repetitions per sequence or one repetition per sequence, a motif size between 50 and 150 aa, and a maximum of 25 motifs.

### 4.5. Real-Time Quantitative Polymerase Chain Reaction (qPCR) and Tissue Distribution Analysis, as Well as Determination of Immune Response to Different Stimuli

For the study, a control group of healthy lamprey larvae was selected and injected with PBS every other day on the 1st, 3rd, and 5th days, with tissue collection taking place on the 6th day. The concentration of PBS, PHA, and LPS was 100 μg/mL. Three healthy and non-stimulated *L. reissneri* were selected along with three lampreys stimulated with PBS, PHA, and LPS. Prior to dissection for tissue extraction, the fish were anesthetized and dissected under sterile conditions. Heart, gills, intestine, kidney, and liver tissues were extracted, placed into sterile and RNase-free centrifuge tubes, and immersed in 1 mL of Sample Protector for RNA/DNA. Total RNA extraction was performed using the EasyScript^®^ One-Step gDNA Removal and cDNA Synthesis SuperMix (Takara, Beijing China) reverse transcription PCR kit.

Using the nucleotide sequences of *Lr.* Notch1 and glyceraldehyde-3-phosphate dehydrogenase (GAPDH) from the *L. reissneri* database as templates, primers were designed within their functional domains using PrimerPremier 5.0. The primer designs were then submitted to Shanghai Biotech for subsequent qPCR validation.

Fluorescence real-time quantitative polymerase chain reaction (RT-qPCR) was used to determine the expression of *Lr.* Notch1 in various tissues and the immune response to different stimuli. Amplification was performed using a 7500 Fast Real-Time PCR system with the following parameters: initial denaturation at 95 °C for 30 s to activate the DNA polymerase, followed by 40 cycles of 5 s at 95 °C, 30 s at 60 °C, and 30 s at 72 °C. *Lr*. GAPDH was used as an internal control. Each reaction volume was 20 μL, including 2 μL cDNA (50 ng/μL), 0.4 μL reverse primer, 0.4 μL forward primer, 10 μL 2 × ChamQ Universal SYBR qPCR Master Mix (Vazyme, Nanjing, China), and 7.2 μL ddH2O. All samples were repeated three times.

### 4.6. siRNA Target Design, Injection, and Silencing Validation

The EGF-like domain of the *Lr. Notch1* gene was selected as the target for siRNA design, using the primer sequences provided in Table S4. The design and synthesis of siRNA were entrusted to GenePharma (Shanghai, China). The specific siRNA sequence is shown in Table S4.

Twelve healthy larvae, weighing approximately 3–5 g, were selected for the study. They were treated with siRNA at a concentration of 53.67 μg/mL [1] via intraperitoneal injection, with a dose of 30 μL/g body weight. The negative control group (NC) underwent a treatment similar to that of the experimental group. After 48 h, 96 h, and 120 h post-injection, the heart tissues of the lamprey larvae were collected following anesthesia. The tissues were rapidly frozen in liquid nitrogen and stored at −80 °C for future use.

RNA extraction was performed on the collected heart tissue, followed by reverse transcription into cDNA using the same aforementioned methods. The subsequent silencing efficiency was validated by combining the synthesized primers with qPCR technology.

### 4.7. Transcriptome Sequencing and Data Analysis

To investigate the impact of *L. reissneri* silencing on heart tissues, transcriptome sequencing was performed. The experimental group, with *L. reissneri* silencing for 48 h, and the control group were compared using three biological replicates. The analysis focused on mRNA, circular RNA, and long noncoding RNA to obtain differential expression profiles. A total of 431 differentially expressed genes were identified, consisting of 169 upregulated genes and 262 downregulated genes. The selection criteria included a *p* value < 0.05 and a fold change ≥ 2. To gain insights into the functional significance of these genes, gene enrichment analysis of Gene Ontology (GO) terms and Kyoto Encyclopedia of Genes and Genomes (KEGG) pathways was performed using the ClusterProfiler package. Based on the results of the enrichment analysis, the differentially expressed genes were further screened and summarized. Additionally, 10 differentially expressed genes were randomly chosen for quantitative polymerase chain reaction (qPCR) validation to ensure the reliability of the transcriptome data.

### 4.8. Statistical Analysis

The bar chart analysis in this study entailed the utilization of three independent replicates of pre-existing data. Standard deviation was computed, and confidence intervals (mean ± standard deviation (SD)) were determined. Additionally, statistical tests, specifically two-tailed *t*-tests, were conducted. Given the notable stress response of larval lampreys to siRNA or stimuli administered into the peritoneal cavity, an initial sample size of 12 was selected. However, a minimum of three samples was consistently ensured, and in cases where the sample size fell below three, the experiment was repeated.

All statistical analyses were carried out using GraphPad Prism 8.0 software. Group differences were assessed through a two-way analysis of variance (ANOVA). A significance threshold of *p* < 0.05 was set (* *p* < 0.05, ** *p* < 0.01, *** *p* < 0.005), with the Bonferroni correction applied for multiple comparisons. The data are presented as the mean ± SD of no fewer than three independent experiments, thereby ensuring the reliability of duplicate samples.

## Data Availability

The data supporting the results of this study are included in this paper. The data obtained in this study and the materials used in this study can be obtained from the corresponding authors according to reasonable requirements.

## Supplementary Materials

The following supporting information can be downloaded at: https://www.mdpi.com/article/10.3390/ijms25010611/s1.
