# Supplementary material for: Exploring the Multiple Roles of Notch1 in Biological Development: An Analysis and Study Based on Phylogenetics and Transcriptomics
Source: Int J Mol Sci. 2024 Jan 3;25(1):611. doi: 10.3390/ijms25010611 (PMC10778765; doi:10.3390/ijms25010611)
Supplement: Supplementary file 1 [file ijms-25-00611-s001.zip › Table S1 The Notch protein accession numbers in all examined species.pdf]

Table S1 The Notch protein accession numbers in all examined species

Species	Symbol	Accession No.	Species	Symbol	Accession No.
Homo sapiens	Hs-Notch1	NP_060087.3	Zootoca vivipara	Zv-Notch1	XP_034969040.1
	Hs-Notch2	NP_077719.2		Zv-Notch3	XP_034960669.1
	Hs-Notch3	NP_000426.2		Zv-Notch4	XP_034962932.1
	Hs-Notch4	NP_004548.3	Thamnophis elegans	Te-Notch1	XP_032088657.1
Mus musculus	Mm-Notch1	NP_032740.3		Te-Notch3	XP_032094875.1
	Mm-Notch2	NP_035058.2	Larimichthys crocea	Lc-Notch1	XP_010736162.1
	Mm-Notch3	NP_032742.1	Bombina bombina	Bb-Notch2	XP_053549070.1
	Mm-Notch4	NP_035059.2	Caretta caretta	Cc-Notch2	XP_048717891.1
Oryctolagus cunniculus	Oc-Notch1	XP_051685646.1	Danio rerio	Dr-Notch2	NP_001108566.2
	Oc-Notch2	XP_051713306.1		Dr-Notch3	NP_571624.2
	Oc-Notch3	XP_051693891.1	Perca flavescens	Pf-Notch2	XP_028443686.1
	Oc-Notch4	XP_051710870.1		Pf-Notch3	XP_028454323.1
Gallus gallus	Gg-Notch1	NP_001384725.1	Tachysurus fulvidraco	Tf-Notch2	XP_047679041.1
	Gg-Notch2	NP_001238962.2	Callorhinchus milii	Cm-Notch2	XP_042190840.1
Taeniopygiaguttata	Tg-Notch1	XP_030142444.3		Cm-Notch3	XP_042200591.1
	Tg-Notch2	XP_030135623.1	Morone saxatilis	Ms-Notch3	XP_035510441.1
Xenopus tropicalis	Xt-Notch1	NP_001090757.1	Scyliorhinus canicula	Sc-Notch3	XP_038640942.1
	Xt-Notch2	XP_002939126.3	Petromyzon marinus	Pm-Notch1L-1	XP_032832376.1
	Xt-Notch3	XP_017948007.1		Pm-Notch1L-2	XP_032832377.1
	Xt-Notch4	XP_031747113.1	Lethenteron reissneri	Lr-Notch1	evm.TU.Hic_chr_12.269
Xenopus laevis	Xl-Notch4	XP_031747114.1	Branchiostoma floridae	Bf-Notch1	XP_035684128.1
