# Supplementary material for: Exploring the Multiple Roles of Notch1 in Biological Development: An Analysis and Study Based on Phylogenetics and Transcriptomics
Source: Int J Mol Sci. 2024 Jan 3;25(1):611. doi: 10.3390/ijms25010611 (PMC10778765; doi:10.3390/ijms25010611)
Supplement: Supplementary file 1 [file ijms-25-00611-s001.zip › Table S2 The conserved motifs discovered among the amino acid sequences of the Notch gene families from vertebrates and invertebrates using the MEME system.pdf]

Table S2 The conserved motifs discovered among the amino acid sequences of the *Notch* gene families from vertebrates and invertebrates using the MEME system.

Number	Motif number	Number of amino acids	Best possible match
2	2	106	TCVDGVNTYNCQCPPEWTGQYCTEDVDECQLQPNACQNGGTCFNTHGGYNCVCVNGWTGEDCSENIDDCATAACFNGATCHDRVASFYCECPHGKTGLLCHLDDAC
	3	138	NPCEHGGKCVNTZGSFQCQCLRGYTGPRCETDINECLSNPCQNDATCLDRIGEFTCICMPGFEGVYCEIDIBECQSNPCLNNGVCLDKVNGFQCECPPGFTGSMCQIDIDECASTPCKNGAKCVDRPNGYECECAEGF
4	4	110	GWTGLYCDVPNVSCEVAASQRGVDVEQLCQHGGLCVBAGNTHYCQCPAGYTGSYCEEZVDECSSNPCQNGATCRDYLGGYSCECVPGYQGVNCEYEIBECQSQPCQNGGT
5	5	106	CLPGFTGPRCZTEINECASNPCKNGGTCTDYVNSYTCTCPPGFSGIHCENNIPDCTESSCFNGGTCVDGINSFTCLCPPGFTGSYCQHEIBECDSQPCLNGGTCVD
6	6	70	SANVISDLIYQGASLQAQTDRTGETALHLAARYARADAAKRLLDAGADANAQDNMGRTPLHAAVAADAQG
7	7	98	DGIASFTCLCQPGYTGYRCENQINECHSNPCQNGGKCIDLVNGYLCNCLPGTSGVNCEINFDDCASNPCDYGVCIDGINRYDCVCEPGFTGPMCNVEI
8	1	98	VFQILIRNRATDLDARMNDGTTPLILAARLAVEGMVEELINCHADVNAVDDLGKSALHWAAAVNNVEATLVLLKNGANKDMQDNKEETPLFLAAREGS
	8	127	PCHNGGTCVDGVNGFTCLCPEGFHDPLCYSZVBECNSNPCIHGTCHDDLNGYKCDCDPGWVGTNCDVDKNECESNPCQNGGTCKDLVNGYRCTCREGFKGPNCQVNINECASNPCLNQGTCIDGVAS
	12	52	YEAAKVLLDHFANRDITDHMDRLPRDIAQERMHHDIVRLLDEYNLVRSPQGH
	13	70	DDVDDRQWTQQHLLAADIRRPPSMALTPPQGEQDADCMDVNVRGPDGFTPLMLASCRGGGLETGNSEEEE
9	9	98	CVDRVGGYSCNCPPGFVGERCEGDVNECLSNPCDPRGTLDCVQLVNDYQCVCRPGYTGRRCESVIBGCESQPCQNGGTCAVASNTPLGFTCKCPPGFS
10	10	90	QEKQCNPLYDKYCADHFADGHCDQGCNNEECGWDGLDCANEVPERLADGTLVLVVLLPPEELLRDSTNFLRELSSLLHTNLRFKLDANGQ
11	11	70	GGTCINTPGSYRCQCPPGYTGQHCESPYVPCAPSPCLNGGTCRQTGDTTYECACLPGFEGQNCEENIDDC
14	14	70	DYESFSCLCPPGWQGQTCEIDIBECVSSPCRNGGTCTNTLGSYRCLCRPGYTGRBCETDIBDCSPNPCLN
15	15	86	NRQCNQQCSZCFDSAFDAAAALGALAALGALNIPYPIPAVSSESEEPPPPSQPLLYLVAVAALJJLVILGLGVLVARRKREHGTLW
16	16	69	JAECAEKAGDGVCDRECNTHACGWDGGDCSLNVDDPWKNCSSSLQCWLYFNBGCCDPCCNSECCLDBGF
17	17	75	YCCCCPLGFGGECCLTPLDNCCLSSPCRNGGTCHLLSLGEYKCRCPPGWTGKTCQLADPCASNPCANGGTCTSTE
18	18	70	SQHSSSSPDDTPKHHLHLPSEHPYLTPSPESPDQWSSSSPHSNSDWSDSTPSPPPTGMQSQQAHIPEAQK
19	19	70	KKARQGKKKRKPSSKGLSAGKKSKKAKKRRKKLSLDGKGQLLESSVTLSPVDSLESPHGYLSBPASPPLM
20	20	50	GFFKRRESSNKKRREPVGZDAVGLKNLKKASDEDLLDDNQNEEHDEDDEG
21	21	70	LLLLLLLPALAAGLQCRDQPEPCLNGGTCETYSNGTGYCKCPPGFVGEYCQLPBPCLSSPCKNGGTCSAV
22	22	68	NGGGCRCTCRGARCRCSPGFGGPECQGPSNSGCASNPCYNGGTCQPTPQPPFYRCLCPPGFTGLRCEI
23	23	92	GGPMNFSVGRVSPVNGQCEWLSRLZSSMVQQQYMVLRVAVQPGGHPQQQQALQHGMLLPLHMGVSRESLPPIVTFQLPPKGSIQQQALPQQQ
24	24	70	VNGTVQYTCSCPRGFRGQDCSLIDACASSPCANGARCTNWNNRYNCTCPPGYQGKNCRTDIDECRRPGLC
25	25	50	SMYQIPDLAQLPSSSFPVATIPPQDGQVLQTSLPSYHQFPMSTGKYPTPP
