# Supplementary material for: Exploring the Multiple Roles of Notch1 in Biological Development: An Analysis and Study Based on Phylogenetics and Transcriptomics
Source: Int J Mol Sci. 2024 Jan 3;25(1):611. doi: 10.3390/ijms25010611 (PMC10778765; doi:10.3390/ijms25010611)
Supplement: Supplementary file 1 [file ijms-25-00611-s001.zip › Table S3 Lr-Notch1 PCRQ-PCR Primer.pdf]

Table S3 *Lr-Notch1* PCR/Q-PCR Primer

Gene name	Gene ID	PCR/RT-PCR	F-Primer	R-Primer
Lr-Notch1	Chr.12.269	PCR	GGGGCCCATGTGTCAGATCGA	AAGGAGGACAAGTTCACCGAC
		RT-PCR	ACTGAAGGTGTGAACTGTGAGACG	TGACGCCATCCACGCACTTC
