# Supplementary material for: Exploring the Multiple Roles of Notch1 in Biological Development: An Analysis and Study Based on Phylogenetics and Transcriptomics
Source: Int J Mol Sci. 2024 Jan 3;25(1):611. doi: 10.3390/ijms25010611 (PMC10778765; doi:10.3390/ijms25010611)
Supplement: Supplementary file 1 [file ijms-25-00611-s001.zip › Table S4 siRNA sequences..pdf]

Table S4 siRNA sequences

Gene name	Sense（5'-3'）	Antisense（5'-3'）
Notch1-1	GCGAGCUGAACGUUAACGATT	UCGUUAACGUUCAGCUCGCTT
Notch1-2	GUGCUACUCAGAGACUGAUTT	AUCAGUCUCUGAGUAGCACTT
Notch1-3	GCCAGAAGAAUGUGAACGATT	UCGUUCACAUUCUUCUGGCTT
Notch1-4	GUAACUGUGAGGCGGUGAUTT	AUCACCGCCUCACAGUUACTT
Negative Control	UUCUCCGAACGUGUCACGUTT	ACGUGACACGUUCGGAGAATT
