# Supplementary material for: Exploring the Multiple Roles of Notch1 in Biological Development: An Analysis and Study Based on Phylogenetics and Transcriptomics
Source: Int J Mol Sci. 2024 Jan 3;25(1):611. doi: 10.3390/ijms25010611 (PMC10778765; doi:10.3390/ijms25010611)
Supplement: Supplementary file 1 [file ijms-25-00611-s001.zip › Table S5 Q-PCR Primer..pdf]

Table S5 Q-PCR Primer

Gene name	Gene ID	F-Primer	R-Primer
GAPDH	chr.27.277	GCTGACAGGCATGGCGTTCC	GGATTCCCTTCAGTTCACCCTTGG
C1qA	chr_69.72	GACGAGGTGTGGCTGGAGATG	GTACAAGATATAGCCGCTGAACACG
P38	chr_30.86	CAGTCGGTCATCCACGCCAAG	GATACAGGTCGTCAAACTCCTCCAG
Wnt1	chr_54.90	GCGGCGACAACCTGGAGTAC	CTGTTGCTGCTGCTGCTGTTG
GNA13	chr_44.85	TTGTAGGCTCGCCGCATCAG	CGGTGGTGAAGTGGTGGAAGAG
VLRA	chr_32.161	AGTGGTTGAGGCTGGACTACAATC	TCGTGGGAAGTGGTCAAAGGTG
VLRB	chr_2.94	CATCCTTCTCATCCTCCTCGTCATC	CGCCACCTCTGCTGTCTCAG
TLE3	chr_24.290	GCGAGCGAGAAGACGGAGATG	CTGCGACAGGAACGGGATGAC
NFKB1	chr_47.213	ATCGTTCTTGAGAGCGGCTTCC	CAGGTAGGCGGTGAACATGAGG
HES5	chr_36.81	AAGCAGCAGCAGAGCAGCAG	CCAGCACATCCGCCTTCTCC
PPARD	chr_34.249	CTACCTCCGCATATTCCCACTGTC	TTGTGCCGTAACCCGACTTCTG
