# Supplementary material for: Exploring the Multiple Roles of Notch1 in Biological Development: An Analysis and Study Based on Phylogenetics and Transcriptomics
Source: Int J Mol Sci. 2024 Jan 3;25(1):611. doi: 10.3390/ijms25010611 (PMC10778765; doi:10.3390/ijms25010611)
Supplement: Supplementary file 1 [file ijms-25-00611-s001.zip › Table S6 Notch signaling pathway related molecules.pdf]

Table S6 Notch signaling pathway related molecules

Gene name	Gene ID	Gene name	Gene ID
Notch	chr_12.269	CTBP	chr_17.1
Delta	chr_25.68	SMRT	chr_28.129
Jagged	chr_8.21	CIR	chr_31.154
ADAM10	chr_1.205	HES1	chr_37.3
ADAM17	chr_29.20	HES5	chr_36.81
PSEN	chr_25.47	NFKB1	chr_47.213
CSL	chr_10.74	HEY	chr_19.249
HDAC	chr_19.169	APH	chr_50.30
