# Supplementary figures and images for: Exploring the Multiple Roles of Notch1 in Biological Development: An Analysis and Study Based on Phylogenetics and Transcriptomics
Source: Int J Mol Sci. 2024 Jan 3;25(1):611. doi: 10.3390/ijms25010611 (PMC10778765; doi:10.3390/ijms25010611)

Volcano Plot of Notch1 p-value<0.8 && |log<sub>2</sub> FC|>0

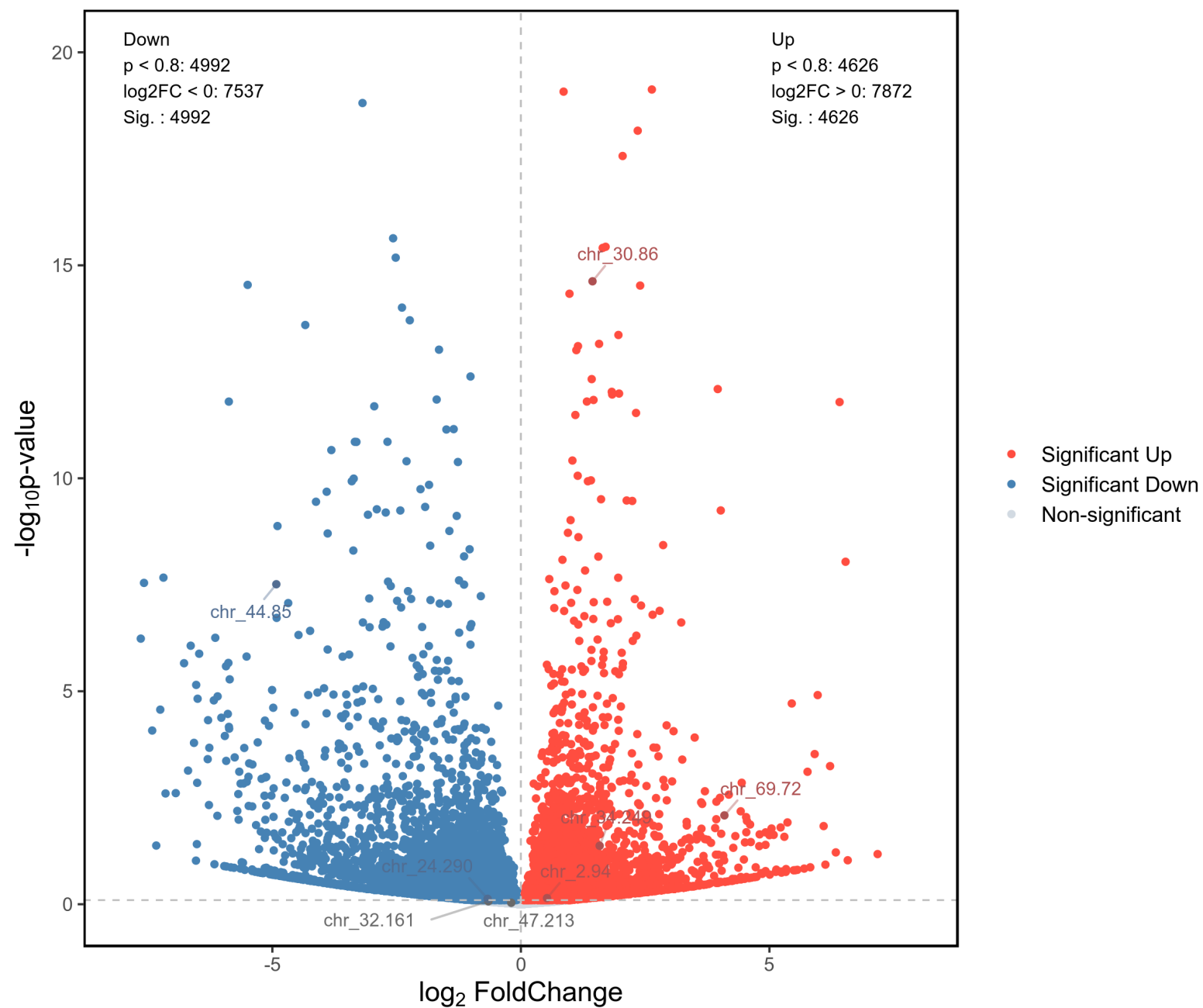

Supplement: Supplementary file 1 [file ijms-25-00611-s001.zip › Figs. S1 Volcano plot of differential genes in Lr. Notch1 silencing transcriptome.pdf]
